# Predicting complications of major head and neck oncological surgery: an evaluation of the ACS NSQIP surgical risk calculator

**DOI:** 10.1186/s40463-018-0269-8

**Published:** 2018-03-22

**Authors:** Peter S. Vosler, Mario Orsini, Danny J. Enepekides, Kevin M. Higgins

**Affiliations:** Department of Otolaryngology—Head & Neck Surgery, Sunnybrook Health Sciences Centre, University of Toronto, 2075 Bayview Avenue, Suite M1 102, Toronto, ON M4N 3M5 Canada

**Keywords:** National surgical quality improvement program risk calculator, Risk assessment, Head and neck, Cancer, Brier score, Surgical complications, Outcome measures, Free flap reconstruction, Microvascular reconstruction

## Abstract

**Background:**

The American College of Surgeons National Surgical Quality Improvement Program (ACS NSQIP) universal surgical risk calculator is an online tool intended to improve the informed consent process and surgical decision-making. The risk calculator uses a database of information from 585 hospitals to predict a patient’s risk of developing specific postoperative outcomes.

**Methods:**

Patient records at a major Canadian tertiary care referral center between July 2015 and March 2017 were reviewed for surgical cases including one of six major head and neck oncologic surgeries: total thyroidectomy, total laryngectomy, hemiglossectomy, partial glossectomy, laryngopharyngectomy, and composite resection. Preoperative information for 107 patients was entered into the risk calculator and compared to observed postoperative outcomes. Statistical analysis of the risk calculator was completed for the entire study population, for stratification by procedure, and by utilization of microvascular reconstruction. Accuracy was assessed using the ratio of predicted to observed outcomes, Receiver Operating Characteristics (ROC), Brier score, and the Wilcoxon signed–ranked test.

**Results:**

The risk calculator accurately predicted the incidences for 11 of 12 outcomes for patients that did not undergo free flap reconstruction (NFF group), but was less accurate for patients that underwent free flap reconstruction (FF group). Length of stay (LOS) analysis showed similar results, with predicted and observed LOS statistically different in the overall population and FF group analyses (*p* = 0.001 for both), but not for the NFF group analysis (*p* = 0.764). All outcomes in the NFF group, when analyzed for calibration, met the threshold value (Brier scores < 0.09). Risk predictions for 8 of 12, and 10 of 12 outcomes were adequately calibrated in the FF group and the overall study population, respectively. Analyses by procedure were excellent, with the risk calculator showing adequate calibration for 7 of 8 procedural categories and adequate discrimination for all calculable categories (6 of 6).

**Conclusion:**

The NSQIP-RC demonstrated efficacy for predicting postoperative complications in head and neck oncology surgeries that do not require microvascular reconstruction. The predictive value of the metric can be improved by inclusion of several factors important for risk stratification in head and neck oncology.

## Background

Identifying patients at increased risk for perioperative complications imperative for determining candidates for surgical intervention and proper preoperative patient counselling. This is particularly relevant in the head and neck oncology discipline where complications such as fistula development and free flap necrosis can result in significantly extended length of stay and decreased quality of life. Surgery-specific risk calculators are available for various surgical practices [1–4], and risk factors for cardiovascular and head and neck-specific complications for head and neck procedures including duration of anaesthesia and ASA are known [5, 6]; however, there are no head and neck-specific risk calculators available.

The universal surgical risk calculator created by the American College of Surgeons National Surgical Quality Improvement Program (ACS NSQIP) was developed as both a surgical aid and informed consent tool to improve the overall decision-making process. The risk calculator is an open-access online tool that uses an algorithm and validated data from over 500 hospitals and 2.7 million operations performed in the Unites States to predict the likelihood of 12 postoperative outcomes [7–11]. This online program accepts the input of 20 comorbidity and demographic-related, patient-specific variables, (such as age group, gender, smoking status, functional status, body-mass index, etc.) in conjunction with a surgery-specific Current Procedure Terminology (CPT) code, to predict patients’ risk of 12 postoperative outcomes within 30 days after surgery [12, 13]. Patient-specific variables chosen for the risk calculator are broadly applicable for patients undergoing one of the 1887 procedures covered [11, 13, 14].

Recently, this system has been evaluated in several surgical subspecialties including head and neck surgery [8, 15–17], urology [9], neurology [10], pulmonology [18], gynecology [19], gastroenterology [12, 20–23], orthopedics [24] and general surgery with variable results. Despite these numerous publications, very few studies have examined the risk calculator’s efficacy in a Canadian setting [13, 14] and none, so far, have evaluated head and neck surgery in a Canadian setting. We aim to provide the first comprehensive evaluation of the risk calculator for head and neck surgery in a Canadian setting by assessing its accuracy in predicting complications following six major head and neck procedures.

Our study evaluates the ACS NSQIP surgical risk calculator for predictive accuracy by comparing forecasted 30-day postoperative outcomes to observed incidences for patients who underwent one of six head and neck surgeries: total thyroidectomy, total laryngectomy, hemiglossectomy, partial glossectomy, laryngopharyngectomy and composite resection. These representative procedures were chosen for review because they are frequently performed by head and neck surgeons (thyroidectomy) or are associated with increased complexity and resultant higher complication rates and average length of stay (composite resection, laryngopharyngectomy). Separate statistical analyses were completed for the overall study population, and for each individual procedure. Furthermore, stratification and statistical analysis by free flap utilization, where patients were either separated into the Free Flap reconstructed (FF) group or Non-Free Flap reconstructed (NFF) group, was completed. This sub-stratification was warranted due to the risk calculator lacking separate CPT codes for head and neck procedures that included free flap reconstruction. This is an important distinction because it is well-established that free flap reconstruction increases patients’ time under anesthesia, and it increases risk of surgical site infection and donor site complications [5, 25, 26].

## Methods

### Study design

A retrospective review of all patients that underwent head and neck surgery at a single Canadian tertiary care referral center, between July 2015 and March 2017 was completed following research ethics board approval. Procedures included for review were total thyroidectomy, total laryngectomy, hemiglossectomy, partial glossectomy, laryngopharyngectomy and composite resection. Each patient’s demographic-, procedure-, and complication- related data were collected and maintained in a secure database.

Demographic information was manually entered into the ACS NSQIP surgical risk calculator [25] to fill 20 patient-specific risk factors including age group, gender, functional status, BMI, ASA class, hypertension, smoking status and COPD. The most relevant Current Procedural Terminology (CPT) codes were then selected based on the type, extent, and attributes of the procedure; the following guidelines were used, hemiglossectomy = 41135, partial glossectomy = 41120, laryngectomy = 31365, laryngopharyngectomy with free flap = 31395, composite resection with free flap = 41153 or 41155, thyroidectomy with central neck dissection = 60252, thyroidectomy without neck dissection = 60240. To maintain consistency, “Surgeon Adjustment of Risk” was not altered.

Patient-specific estimates of postoperative risk, determined by the risk calculator, including serious complications, any complications, pneumonia, cardiac complications, surgical site infection (SSI), urinary tract infection (UTI), venous thromboembolism (VTE), renal failure, readmission, discharge to nursing or rehabilitation facility, return to operating room (ROR), and death, were recorded. Complications were simply tallied, with no additional weight given to multiple complications in the same patient. Observed incidences of complication as determined by database review were compared  with the risk calculator’s predictions.

### Statistical analysis

Statistical analyses were completed for the overall study population, as well as for subset analysis by procedure type, and by presence or absence of free flap reconstruction (FF group and NFF group, respectively). Predicted and observed rates of incidences were then compared within each stratification. Brier score, ROC curves, likelihood ratios and Wilcoxon signed–ranked tests for nonparametric data were generated with SPSS, version 24.0.

### Discrimination - ROC curves

Evaluation of the ACS NSQIP surgical risk calculator for discrimination ability was completed using area under (AUC) the receiver operating characteristics (ROC) curve, sometimes called c-statistic. Receiver operating characteristics curve graphs model sensitivity (true positive predictions) against 1 – specificity (false positive predictions) to evaluate how well a model distinguishes between higher and lower risk units within a population. The area under this graph is considered an accurate representation of a model’s discrimination and is scored between 1.0 to 0.5, where the former is considered perfectly predictive and the latter is equivalent to chance. C-statistic values > 0.7 are considered adequately discriminative whereas values of > 0.8 are considered strongly discriminative [9, 10, 26, 27].

### Calibration - brier score

Calibration is a measure of how well a model’s predictions ‘fit’ an observed incidence rate over a collection of predictions [10]. The Brier score, which is the sum of the mean squared differences between predicted values and binary outcomes, was used to evaluate the ACS NSQIP surgical risk calculator for calibration. Incidences were given a value of 1 and non-incidences were given a value of 0. Brier score values range from 0.0 to 1.0, where smaller values represent increased calibration and accuracy. Several thresholds, varying from 0.01 to 0.16, have been suggested to quantify sufficient accuracy for the Brier score [16, 17, 19, 21]; 0.09 was used as it is considered accurate for outcomes with low incidences [28, 29].

## Results

### Patient characteristics

Patients that underwent operations at a single, tertiary, Canadian teaching hospital between July 2015 and March 2017 were retrospectively reviewed for the purposes of this study. A total of 131 patients underwent operations, and 24 patients lacking adequate records or without 30-day follow-up information were excluded from the study, leaving a final population size of 107 individuals. Twenty-seven postoperative complications were observed within 30 days of surgery for the patients in this study (25%). The patient group had a mean age of 61 years, was composed approximately evenly of males and females (54, 53 years, respectively) and was, on average, overweight (Body Mass Index = 27.6 mg/kg^2^). Most patients were non-diabetic (87%) and exhibited a high incidence of hypertension (40%). There were no emergency cases (0%), and one patient was on dialysis before their operation (1%). The most commonly observed postoperative complications were ROR (12 incidences, 11%), SSI (11 incidences, 10%) and readmission (5 incidences, 5%). A full outline of demographic information can be found in Table 1.

**Table 1:** Demographic and Medical Information

Characteristic	Value (*n* = 107)
Age, mean (SD), y	61.1 (13.4)
Male gender, # (%)	54 (50)
Height, mean (SD), m	1.65 (0.14)
Weight, mean (SD), kg	74.1 (17.2)
BMI, mean (SD)	27.6 (6.65)
Underweight, # (%)	5 (5)
Normal weight, # (%)	29 (27)
Overweight, # (%)	29 (27)
Obese, # (%)	23 (21)
Unknown, # (%)	21 (20)
Functional Status, # (%)	
Independent	106 (99)
Partially Dependent	1 (1)
Dependent	0 (0)
ASA Class, # (%)	
I	26 (24)
II	54 (50)
III	27 (25)
IV	0 (0)
Diabetes, # (%)	
No	93 (87)
Oral Medication	4 (4)
Insulin	10 (9)
Hypertension requiring medication, # (%)	
Yes	43 (40)
No	64 (60)
Dyspnea, # (%)	
No	92 (86)
With moderate exertion	12 (11)
At rest	3 (3)
Current Smoker (within 1 year), # (%)	
Yes	23 (21)
No	84 (79)
History of severe COPD, # (%)	
Yes	13 (12)
No	94 (88)
Dialysis, # (%)	
Yes	1 (1)
No	106 (99)
Steroid use for chronic condition, # (%)	
Yes	8 (7)
No	99 (93)
Emergency case: Yes, # (%)	0 (0)
Ascites within 30 days prior to surgery: Yes, # (%)	0 (0)
Sepsis within 48 h: Yes, # (%)	0 (0)
Acute renal failure: Yes, # (%)	0 (0)
Use of mechanical ventilation: Yes, # (%)	0 (0)
Congestive Heart Failure 30 days prior to surgery: Yes, # (%)	0 (0)

### Predicted vs. observed outcomes

**Fig. 1:**
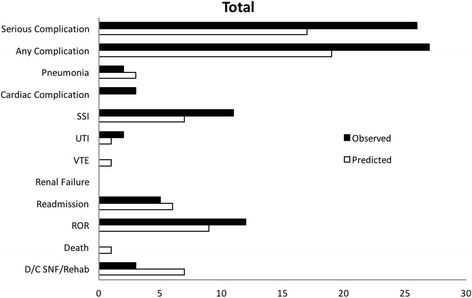
Predicted vs. observed incidences for total study population

Summary analyses demonstrating total observed and predicted incidences of complications without stratification were calculated (Fig. 1). The risk calculator exhibited 100% accuracy in predicting renal failure and adequate performance in predicting readmission (predicted = 6, observed = 5) and ROR (predicted = 9, observed = 12). However, it underestimated serious complications (35%), any complication (30%), cardiac complications (100%), SSI (36%), UTI (50%), and ROR (25%). Overestimations were equally common with pneumonia (50%), VTE, readmission (20%), death, and discharge to nursing and rehabilitation (133%), all scoring below predicted.

**Fig. 2:**
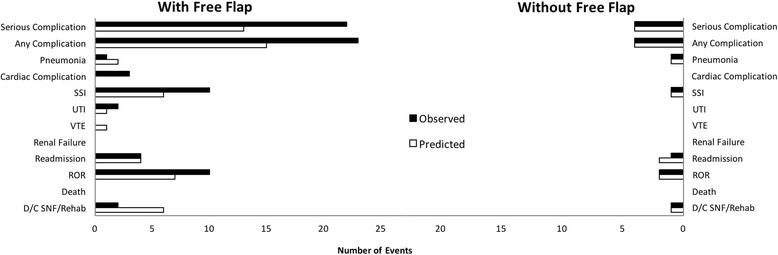
Predicted vs. observed incidences by free flap reconstruction

Stratification of the data based on use of free flap reconstruction revealed that the NSQIP- risk calculator was accurate in patients that did not receive microvascular reconstruction. Figure 2 depicts the stratification of predicted complications as calculated by the ACS NSQIP surgical risk calculator compared to observed incidences for those patients who underwent free flap reconstruction (*n* = 58) and those that did not (*n* = 49). The risk calculator correctly predicted the number of complications in the NFF patient group for 11 of 12 outcomes, but exhibited a 100% overestimation for the remaining outcome—readmission (predicted = 2, observed = 1). For the patient cohort that underwent free flap reconstruction, the risk calculator demonstrated more limited accuracy, predicting only renal failure (predicted = 0, observed = 0), readmission (predicted = 4, observed = 4) and death (predicted = 0, observed = 0), within acceptable error. Six of the remaining nine outcomes were underestimated, while the rest were overestimated.

### Free flap vs. no free flap

In our population, 58 (54%) patients underwent free flap reconstruction as part of their procedure. C-statistic and Brier score analyses were calculated for each of these stratifications. Overall, the risk calculator demonstrated improved calibration and comparable discrimination in predicting complications for the non-free flap reconstructed group when compared to the free flap reconstructed group (Table 2). Values shown in bold are considered to meet or exceed the threshold values for their respective test statistics. Twelve of twelve Brier score values in the NFF group were below threshold (0.09) and 3 of 12 c-statistic values reached adequacy (0.70). Conversely, in the FF group, only 8 of 12 Brier score values showed acceptable calibration and 4 of 12 c-statistic values scored above 0.70. Specifically, SSI (Brier = 0.019, ROC = 0.885), readmission (Brier = 0.018, ROC = 1.00), and discharge to nursing or rehabilitation (Brier = 0.023, ROC = 0.854) exhibited adequate discrimination and calibration in the NFF group, and pneumonia (Brier = 0.017, ROC = 0.912), cardiac complication (Brier = 0.051, ROC = 0.767), readmission (Brier = 0.063, ROC = 0.713), and discharge to nursing or rehabilitation (Brier = 0.028, ROC = 0.987) scored adequately in the FF group. C-statistic values shown as ‘N/A’ were incalculable due to an absence of observed complications for those outcomes.

**Table 2:** Brier score and ROC AUC by Risk Calculator outcome

	Without Free Flap^*,†,a^		With Free Flap^*,†,a^		Combined^*,†,a^	
Outcome	Brier Score	ROC	Brier Score	ROC	Brier Score	ROC
Serious Complications	**0.0838**	0.5220	0.2508	0.6060	0.1743	**0.7070**
Any Complications	**0.0861**	0.5500	0.2474	0.6280	0.1735	**0.7190**
Pneumonia	**0.0208**	0.3230	**0.0171**	**0.9120**	**0.0188**	0.5430
Cardiac Complications	**0.0000**	N/A	**0.0509**	**0.7670**	**0.0276**	**0.8380**
SSI	**0.0193**	**0.8850**	0.1453	0.6830	**0.0876**	**0.7900**
UTI	**0.0001**	N/A	**0.0339**	0.4420	**0.0184**	0.4980
VTE	**0.0001**	N/A	**0.0002**	N/A	**0.0001**	N/A
Renal Failure	**0.0000**	N/A	**0.0000**	N/A	**0.0000**	N/A
Readmission	**0.0183**	**1.0000**	**0.0627**	**0.7130**	**0.0445**	0.6040
ROR	**0.0234**	0.2190	0.1406	0.6880	**0.0869**	**0.7500**
Death	**0.0000**	N/A	**0.0001**	N/A	**0.0001**	N/A
D/C SNF/Rehab	**0.0232**	**0.8540**	**0.0281**	**0.9870**	**0.0259**	**0.8510**

*Brier scores of < 0.09 and ROC > 0.7 (bold values) were considered accurate

^a^N/A indicates no ROC score could be calculated due to the absence of complications

^†^Bold values meet or exceed their respective accuracy thresholds

When considering the entire study population without stratification by free flap, the risk calculator benefited from the effects of increased heterogeneity on discrimination, with 6 of 12 outcomes scoring above 0.70. Outcomes that scored satisfactorily for both discrimination and calibration were: cardiac complication (Brier = 0.028, ROC = 0.838), SSI (Brier = 0.088, ROC = 0.790), ROR (Brier = 0.087, ROC = 0.750), and discharge to nursing or rehabilitation (Brier = 0.026, ROC = 0.851). Two of the remaining outcomes, serious complications and any complications, scored acceptably for discrimination (ROC = 0.707 and 0.719, respectively), but inadequately for calibration (Brier = 0.174 and 0.174, respectively). Conversely, pneumonia, UTI, and readmission met calibration requirements (Brier = 0.019, 0.018, and 0.045, respectively), but did not score above the threshold for discrimination (ROC = 0.543, 0.498, and 0.604, respectively). Incidences of death, venous thromboembolism or renal failure were not observed; thus, c-statistics for these outcomes could not be calculated.

### Stratification by procedure

Evaluation of the risk calculator was also stratified by procedure (Table 3) where patients were separated into one of the following groups: hemiglossectomy with free flap, partial glossectomy, laryngectomy, laryngopharyngectomy with free flap, composite resection with free flap, and thyroidectomy. Thyroidectomy was further subdivided into procedures with central neck dissection and those without. Table 3 indicates the number of patients in each stratum, the percentage of patients with stage IV cancer for cases with available staging information (*n* = 89), and the instances of preoperative radiation. One patient was excluded from analysis as a result of procedural characteristics not adequately fitting into the above groups. The occurrences of preoperative radiation (Likelihood Ratio = 21.689, *p* = 0.003) and the incidences of stage IV cancer (Likelihood Ratio = 49.314, *p* = 0.001) were significantly different between groups. This is to be expected considering the different standards of care associated with different disease locations and extensions. The risk calculator demonstrated adequate calibration and discrimination for laryngopharyngectomy with free flap (Brier = 0.073, ROC = 0.842), composite resection with free flap (Brier = 0.081, ROC = 0.842), thyroidectomy overall (Brier = 0.024, ROC = 0.817), and thyroidectomy with central neck dissection (Brier = 0.023, ROC = 0.816) or without central neck dissection (Brier = 0.027, ROC = 0.845). The risk calculator’s ability to predict complications secondary to laryngectomy showed excellent discrimination (ROC = 0.956) but fell just short of acceptable calibration (Brier = 0.095). The remaining procedures, hemiglossectomy with free flap and partial glossectomy, exhibited acceptable calibration (Brier = 0.003 and 0.001, respectively) but, due to lack of observed complications, had incalculable c-statistics. Overall, the risk calculator performed exceptionally well in predicting complications by procedure; however, this is biased by the few patients in most of the groups.

**Table 3:** Brier Score, ROC AUC, and demographic information by procedure type

Procedure	N	Brier Score^*,†^	ROC^a,†^	% T4a	Radiated Patients	CPT Code(s)
Hemiglossectomy with Free Flap	4	**0.0032**	N/A	25%	0	41,135
Partial Glossectomy	12	**0.0015**	N/A	0%	0	41,120
Laryngectomy	5	0.0954	**0.956**	100%	3	31,365
Laryngopharyngectomy with Free Flap	16	**0.0732**	**0.842**	81%	2	31,395
Composite Resection with Free Flap	39	**0.0807**	**0.842**	30%	4	41,153 & 41,155
Thyroidectomy Overall	30	**0.0241**	**0.817**	7%	0	60,252 & 60,240
Thyroidectomy with neck dissection	21	**0.0229**	**0.816**	7%	0	60,252
Thyroidectomy without neck dissection	9	**0.0273**	**0.845**	0%	0	60,240

*Brier scores of < 0.09 and ROC > 0.7 (bold values) were considered accurate

^a^N/A indicates no ROC score could be calculated due to the absence of complications

^†^Bold values meet or exceed their respective accuracy thresholds

### Overall

The ACS NSQIP surgical risk calculator’s overall discrimination and calibration scores were calculated by evaluating all predictions of complications (12 predictions for each patient) together; this was completed for both FF and NFF groups, as well as, for the entire study population (Table 4). In all three instances the risk calculator exhibited accurate discrimination and calibration: FF (Brier = 0.081, ROC = 0.85), NFF (Brier = 0.023, ROC = 0.78), and combined (Brier = 0.055, ROC = 0.86).

**Table 4:** Brier score and ROC AUC for all complications calculated together

	Without Free Flap^a,b^		With Free Flap^a,b^		Combined^a,b^	
Outcome	Brier Score	ROC	Brier Score	ROC	Brier Score	ROC
Overall	**0.0230**	**0.7790**	**0.0814**	**0.8520**	**0.0548**	**0.8590**

^a^Brier scores of < 0.09 and ROC > 0.7 (bold values) were considered accurate

^b^Bold values meet or exceed their respective accuracy thresholds

### Length of stay

**Fig. 3:**
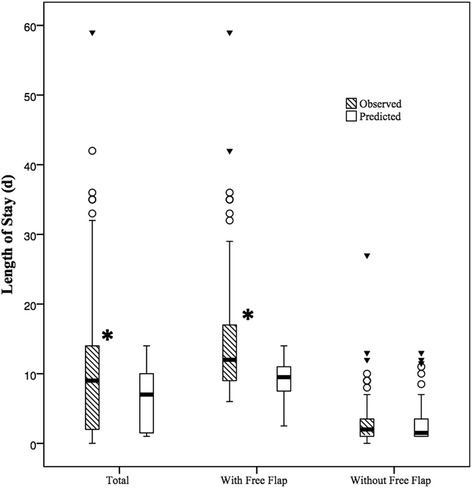
Comparison between predicted and observed length of hospital stay for study population and free flap stratifications. Footnote: *Observed and predicted lengths of stay were found to significantly differ

Length of stay analyses were completed for the overall study population and for both NFF and FF stratifications. Analyses were completed using the Wilcoxon signed–ranked test for nonparametric data [12, 22] and one patient was excluded from analysis due to ongoing hospital stay at time of manuscript development. Figure 3 compares the box plots of observed and predicted lengths of stay for each sub-stratification and the total study population. Predicted lengths of stay for the total study population and for the FF stratified group were found to be significantly different than observed lengths of stay (*p* = 0.001). Conversely, predicted length of stay for the NFF stratified group was not found to be significantly different from observed length of stay (*p* = 0.764).

## Discussion

Improving the informed consent process, providing surgeons with essential preoperative information, and acting as a quality control measure, are all potential benefits obtainable from an accurate surgical risk calculator. For these reasons, and others, various subspecialties have evaluated the efficacy of the ACS NSQIP surgical risk calculator in practice. Results from these studies are markedly inconsistent, with conclusions for the risk calculator ranging from entirely non-predictive for all outcomes [9] to useful within judicious parameters [22].

One potential cause for these varying results is lack of agreement on a threshold value for the Brier Score. Several studies consider scores below 0.01 as predictive [9, 16], referencing Bilimoria et al. and Cohen et al. as justification for this decision. Bilimoria et al., however, does not use a Brier score threshold of 0.01 in their publication and interestingly, considered a value of 0.069 as sufficiently accurate [30]. Additionally, Cohen et al. does not at all use, nor refer to, the Brier score test in their analysis [31]. Other published threshold values are much higher, including 0.16 [21] and 0.14 [22]. Due to these discrepancies, we decided to use 0.09, a relatively intermediate value that has been shown to indicate accuracy for models with low incidence rates [28, 29].

Some studies have found that the surgical risk calculator systemically underestimates the true risk of postoperative complications [20, 32]. Lack of surgery-specific metrics and predicted outcomes inherent to the risk calculator are often implicated as a cause of this inaccuracy [32]. For this reason, many studies have tested the validity of supplementary specialty-specific preoperative metrics to support the 21 already captured by the ACS NSQIP surgical risk calculator program [12, 23, 33]. As opposed to evaluating additional head and neck –specific preoperative metrics, we decided stratification by surgical complexity, free flap reconstruction versus no free flap reconstruction, and surgical type, as was previously unstudied, could delineate the risk calculator's accuracy as a function of surgical complexity. Initial evaluation of the risk calculator for observed versus predicted events, without stratification, exhibited results similar to other studies with 5 of 12 and 6 of 12 outcomes overestimated [9, 23] and underestimated, respectively. However, upon stratification by free flap utilization, the primary driver of these inaccuracies was elucidated. In the FF group, the risk calculator over- or under-predicted 9 of 12 outcomes. In the NFF group, however, outcomes were exceptionally well predicted, with 11 of 12 complications accurately projected by the risk calculator. We believe increased technical difficulty, potential sacrifice of vital structures (i.e. internal jugular vein, spinal accessory nerve, phrenic nerve, sympathetic trunk, and the thoracic duct), longer duration of anaesthesia, and extended post-operative stay, are potential contributors to the disparity between these stratifications.

Statistical results similar to that of predicted and observed outcomes was demonstrated with LOS; length of stay predictions for the overall population and FF sub-group were found to significantly differ from observed postoperative stays. However, when LOS analysis was completed for the NFF stratified group, predicted and observed stays were not found to significantly differ. Furthermore, Fig. 3 clearly depicts the dissimilarity between predicted and observed LOS for the FF group and overall population, when compared to that of the NFF group. Our overall population and the FF sub-group results are consistent with many other publications, which found little to no association between observed and predicted length of stay [12, 15–17, 21, 22]. Massoumi et al. implicated the level of care offered by a hospital (primary vs. tertiary) and its practices as potential generators of these inconsistencies [20].

Our results follow a general trend present in the literature: increasingly complex procedures, where postoperative outcomes are closely influenced by the case-by-case features of the procedure, the skill level of the surgeon and postoperative care practices, are more likely miss-projected by the ACS NSQIP risk calculator, which cannot consider surgical intricacies, surgeon experience or hospital-specific acute care standards [8, 20]. An essential first step in mitigating this inaccuracy, and a function currently missing from the ACS NSQIP surgical risk calculator, is the combination of CPT codes to build a case more representative of the actual procedure [32].

Our analysis of the risk calculator for calibration and discrimination followed a similar trend as LOS with all 12 outcomes properly calibrated in the NFF group and 8 of 12 calibrated in the FF group. As mentioned by Winoker et al., it is important to emphasize that few incidences of observed complications can contribute to low Brier scores [9]. This can perhaps be seen in outcomes such as renal failure, VTE, and UTI in our cohort. Interestingly, the risk calculator exhibited slightly better discrimination for outcomes in the FF group (4 of 12) than in the NFF group (3 of 12). One possible explanation for this phenomenon is the improved performance of models that exhibited higher levels of heterogeneity. As shown by Cohen et al., ACS NSQIP surgical risk calculator estimations, projected for heterogeneous populations of patients (those that undergo many different procedures and have a diversity of preoperative morbidities), score better for discrimination than risk projections for homogenous patient populations [9]. The risk calculator scored well for both calibrated and discrimination for 6 of 12 outcomes in the total study population. Our results demonstrating the risk calculator’s accuracy for a portion of complications predicted are consistent with that of previous publications [21, 22, 33]; however, they are inconsistent with other evaluations specific to the head and neck discipline. Two potential reasons for this discrepancy are differences in Brier score threshold values used and patient population heterogeneity. The former, explained in the discussion above, has resulted in previous studies considering acceptable scores as non-predictive [16, 17]. The latter refers to previous studies evaluating only one head and neck surgical procedure and thus exhibiting lower levels of heterogeneity [15, 16].

Analysis by procedure type further substantiated the risk calculator’s accuracy in our patient cohort, with 7 of 8 evaluated procedures showing good calibration and all calculable procedures meeting adequate discrimination levels. Potential reasons why the risk calculator could have shown poor calibration for laryngectomy, which scored just above the Brier threshold, includes relatively few patients (*n* = 5) having undergone this procedure and thus a potential for sampling error, and the relatively high incidences of T4 cancer (100%) and prior radiation (60%). Interestingly, of the procedures analyzed that met the Brier score threshold, composite resection with free flap and laryngopharyngectomy with free flap had the highest values. This relative decrease in calibration can potentially be attributed to the increased complexity associated with these surgeries. This is certainly supported considering the results of the free flap stratified analyses.

For the overall population analyses, in which all surgical procedures and complications were pooled together, discrimination and calibration computations met their respective thresholds this provides the strongest evidence in our analysis of the risk calculator’s effectiveness in predicting complications secondary to head and neck surgery.

### Primary strengths

We present the first comprehensive evaluation of the ACS NSQIP surgical risk calculator in the head and neck discipline in a Canadian setting. Furthermore, to the authors’ knowledge we are the first publication on the risk calculator to stratify by microvascular reconstruction, a surgical procedure which is associated with longer OR times and increased complication rates. Finally, our findings demonstrate good performance of the ACS NSQIP surgical risk calculator in predicting postoperative complications in patients undergoing head and neck surgery without free flap reconstruction. Given these results, we regularly utilize the risk calculator to determine individual patient’s risk of complications as a decision-support tool to evaluate the safety of the procedure and to provide more comprehensive preoperative counselling to patients and their families.

### Limitations

We are primarily limited by the size of our study cohort (*n* = 107), having few patients with complications (27), and being a single institution retrospective review. The latter two limitations have been implicated by Cohen et al. as potential sources of inaccuracy when evaluating the risk calculator [11]. Specifically, they concluded that three criteria must be met to fairly evaluate the risk calculator: a patient cohort acquired from more than one care center, a minimum of 100 patients with an incidence, and sufficient cohort heterogeneity. Based on the diverse list of procedures reviewed, we believe the level of heterogeneity required has been met in our cohort.

### Next steps

Specialty-specific surgical risk calculators have shown improved predictive accuracy over the ACS NSQIP risk calculator [12, 23, 32], suggesting development of a head and neck-specific risk calculator is warranted. One potential caveat of these additional metrics, as was pointed out by Cohen et al., is the potential for overfitting [11]. Application of surgery-specific risk calculators should be used only on the procedures and settings which they have been examined. To this end, a multicenter collaboration as part of the Canadian Association of Head and Neck Surgeons is planned.

The purpose of a risk calculator is to determine the potential complications our patients may endure based on patient comorbidities and the procedures planned. The ACS NSQIP surgical risk calculator predicts the major complications of any surgery; however, it does not predict complications specific to our patient population that impair quality of life and increase LOS including fistula development and free flap failure requiring a second free flap or locoregional flap. A specialty-specific risk calculator would address this shortcoming.

## Conclusion

The ACS NSQIP surgical risk calculator has the potential to act as a quality improvement metric and aid in the informed consent process through preoperative planning and postoperative prevention of potential mortality and morbidity. Though data collected for the risk calculator was amassed entirely from American hospitals with the intended use of improving the standard of care within the American health care system, our results suggest the potential utility of the risk calculator for Canadian Head and Neck oncology. Specifically, judicious application of the risk calculator for head and neck surgical procedures that do not involve microvascular reconstruction is supported.

## Abbreviations

ACS NSQIP: American College of Surgeons National Surgical Quality Improvement Program
AUC: Area under the curve
COPD: Chronic obstructive pulmonary disease
CPT: Current Procedural Terminology
FF: Free flap reconstructed patient group
LOS: Length of stay
NFF: Non-free flap reconstructed patient group
REB: Research ethics board
ROC: Receiver operating characteristics
ROR: Return to operating room
SSI: Surgical site infection
UTI: Urinary tract infection
VTE: Venous thromboembolism
